# A simple all-fiber comb filter based on the combined effect of multimode interference and Mach-Zehnder interferometer

**DOI:** 10.1038/s41598-018-30213-2

**Published:** 2018-08-07

**Authors:** Guorui Zhou, Rahul Kumar, Qiang Wu, Wai Pang Ng, Richard Binns, Nageswara Lalam, Xinxiang Miao, Longfei Niu, Xiaodong Yuan, Yuliya Semenova, Gerald Farrell, Jinhui Yuan, Chongxiu Yu, Xinzhu Sang, Xiangjun Xin, Bo Liu, Haibing Lv, Yong Qing Fu

**Affiliations:** 1 Laser Fusion Research Center, China Academy of Engineering Physics, Mianyang, 621900 China; 2 Faculty of Engineering & Environments, Northumbira University, Newcastle Upon Tyne, NE1 8ST UK; 3 Photonics Research Centre, Dublin Institute of Technology, Dublin, Ireland; 4 Beijing University of Posts and Telecommunications, Beijing, 100876 China; 5 School of Physics & Optoelectronic Engineering, Nanjing University of Information Science & Technology, Nanjing, China

## Abstract

A polarization-dependent all-fiber comb filter based on a combination effect of multimode interference and Mach-Zehnder interferometer was proposed and demonstrated. The comb filter was composed with a short section of multimode fiber (MMF) fusion spliced with a conventional single mode fiber on the one side and a short section of a different type of optical fiber on the other side. The second type of optical fiber is spliced to the MMF with a properly designed misalignment. Different types and lengths of fibers were used to investigate the influence of fiber types and lengths on the performance of the comb filter. Experimentally, several comb filters with free spectral range (FSR) values ranging from 0.236 to 1.524 nm were achieved. The extinction ratio of the comb filter can be adjusted from 6 to 11.1 dB by varying polarization states of the input light, while maintaining the FSR unchanged. The proposed comb filter has the potential to be used in optical dense wavelength division multiplexing communication systems.

## Introduction

Due to the rapidly increased demand for optical communication systems, the development of novel devices for dense wavelength-division-multiplexing (DWDM) optical networks has attracted considerable attention. Optical fiber comb filters, a key component with compact size and good compatibility with the fiber systems are widely used in the multiwavelength fiber lasers^1^ and optical networks^2^ to process the optical signals and isolate the neighboring channel signals and to reduce the cross-talk. A variety of techniques have been proposed to realize all-fiber comb filter function such as fiber Bragg grating filters^3–5^, Sagnac loop interferometers^6–8^, Fabry–Perot filters^9–11^, birefringent fiber filters^12^ and Mach-Zehnder (M-Z) filters^13–16^. However, these techniques suffer from the disadvantages of high cost and complex fabrication. Comb filters based on a singlemode-multimode-singlemode (SMS) fiber structure offer low cost and easy fabrication and hence have been widely investigated^17–22^. In our previous investigations we have proved that a multimode fiber (MMF) can act as a “mode coupler” to re-couple cladding modes into the singlemode fiber (SMF) and hence to achieve long distance transmission for the cladding modes^23^.

**Figure 1:**
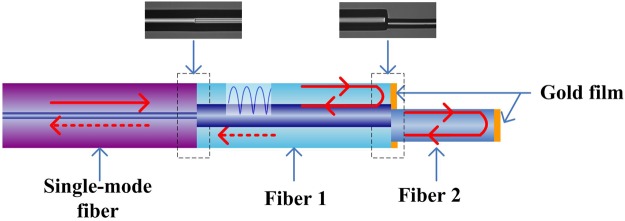
Schematic diagram of the proposed fiber comb filter.

In this paper, a new type of all-fiber comb filter is proposed, based on the “mode coupler” function of the MMF. A schematic diagram of the proposed all-fiber comb filter is shown in Fig. 1.

## Methods

As shown in Fig. 1, the filter consists of three consecutive fiber sections: a conventional SMF, a short section of Fiber 1 (for example, an MMF) and a sort section of Fiber 2 (for example, a small diameter fiber). The SMF is used to transmit the linear polarized light into Fiber 1 and recouple the reflected linear polarized light within Fiber 1. The Fiber 2 is fusion spliced to the end face of the Fiber 1 with a predesigned misalignment. The end faces of both Fiber 1 and 2 are coated with reflective metal films. The light injected from the conventional SMF into Fiber 1 will excite multiple high-order modes in Fiber 1. It is confirmed that interference between these multiple modes within Fiber 1 is dominated. On the end of Fiber 1, optical power is partly coupled into Fiber 2 and partly remained in Fiber 1 on account of the reflectivity of end face. Owing to the fact that an optical path different existing for two beams propagated in Fiber 1 and 2 respectively, a periodic interference spectrum of the in-line MZI is generated in the Fiber 1. And interference light will be re-coupled back to the conventional SMF. As a result, the reflectivity spectrum containing multimode interference and MZI are guided out through the conventional SMF. On highlight of the proposed fiber comb filter should be pointed out that it acts not only fiber comb filter but also a sensor for dual-parameters sensing. This structure could be very sensitive to external refractive index changes so the device must be protected, dust and humidity could produce important changes in the interference pattern.

It should be noted that for the fabrication process, suitable arc time and powers should be carefully selected in order to ensure the integrity of the fiber filter during the fusion splicing process, especially at the splice between Fiber 1 and Fiber 2. In the fabrication process, stepped motors of optical fiber fusion splicer (FITEL, s178A ver.2) were used to accurately control misalignment distance between Fiber 1 and Fiber 2 from side view and top view under the magnified picture on the screen. In order to ensure reliability and repeatability of the fiber filter made by the method, the same arc time and powers and misalignment distance should be used in fabrication process of the fiber filter. The insets in Fig. 1 show the corresponding optical microscope images of the splices of the fiber filter. Gold film is coated on the end face of Fiber 1 and 2 with sputter method in order to improve reflectivity of the proposed fiber comb filter. The selective coating of the fiber with gold film is a key part of the filter fabrication process. The micro-manipulation for the proposed fiber structure was implemented carefully under stereomicroscope (Nikon SMZ1500) in order to cover a thin layer of Polyethylene film ONLY on the side surface of the Fiber 1 and 2. In order to ensure that the film was tightly attached to the side surface of the Fiber 1 and 2, it is necessary to clean the surface of the Fiber 1 and 2 with ethyl alcohol. The sputtering process was postponed until the residual ethyl alcohol evaporated on the end face of the Fiber 1 and 2. After the sputtering, the entire proposed comb filter structure was dipped into ethyl alcohol for about a minute to remove the Polyethylene film coated on the side surface of Fiber 1 and 2.

**Figure 2:**
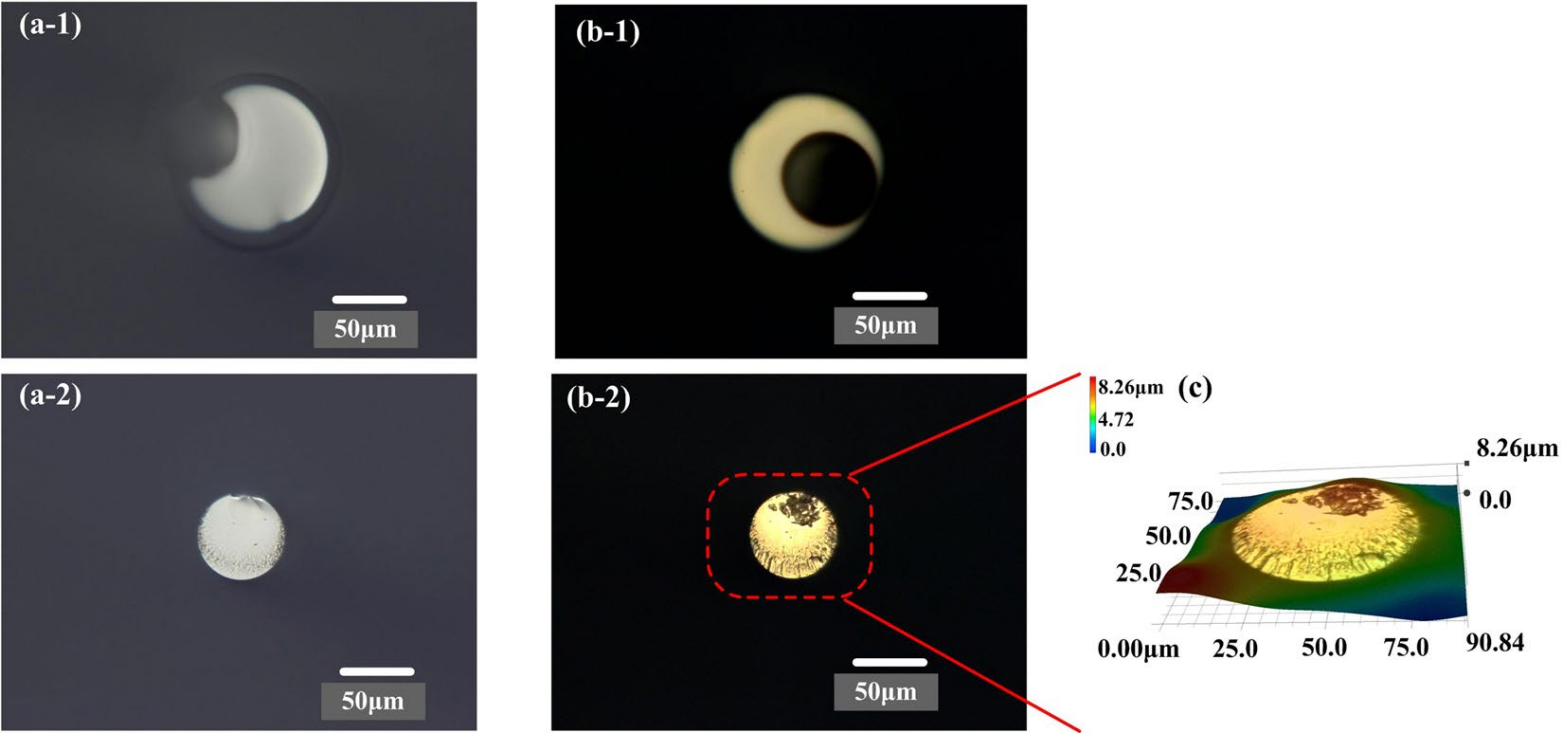
Optical microscope images of fiber cross section without any coating (**a**), with gold film on the end face (**b**) and 3D viewing of Fiber 2 (**c**). Here, the material of Fiber 1 and 2 is NC125 and NC61.5 respectively, (**a-1**) and (**b-1**) are for Fiber 1, (**a-2**) and (**b-2**) are for Fiber 2.

Figure 2 exhibits morphology of the fiber cross section without any coating (a), with gold film on the end face (b) and 3D view of Fiber 2 (c) using optical microscope (Keyence, VHX2000). As shown in inset (a-1),(a-2) and (c), the integrity of the end face of Fiber 1 and 2 is superior, which ensure low insert loss for optical communication systems. At the same time, Fiber 2 stands on the surface of Fiber 1 to form two different branching due to misalignment. From the inset (b-1) and (b-2), gold film was well coated on the end face of Fiber 1 and 2, which will improve reflectivity at the fiber end faces, compared to that without any coating. The inset (c) shows that the roughness of the end face of Fiber 2 is less than 8 μm, which is beneficial to improve the reflectivity on the end face of Fiber 2.

**Figure 3:**
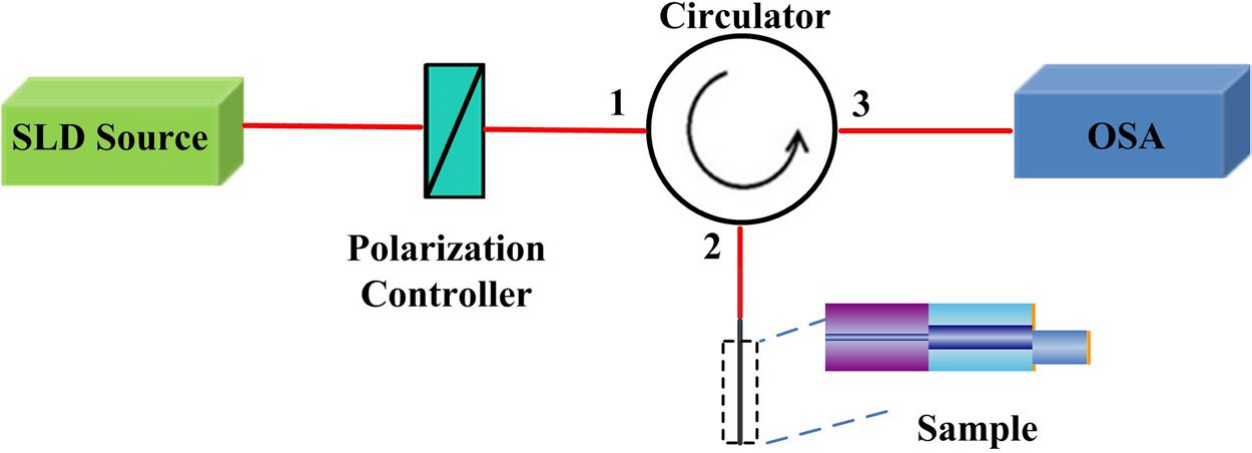
Experimental measurement setup for the fiber comb filter.

Figure 3 illustrates the experimental measurement setup for the proposed comb filter. A superluminescent diode (SLD, Throlabs S5FC 1550P-A2) was used as the optical source with a center wavelength of 1550 nm, which was connected to a polarization controller (PC) before connecting to the Port 1 of a circulator. The sample filter was connected to the Port 2, and Port 3 was connected to a spectrum analyzer to monitor the output signal.

### Data availability statement

The datasets generated during and/or analysed during the current study are available from the corresponding author on reasonable request.

## Results and Discussion

As light incidents from SMF to Fiber 1, the field *E*1(*r*, *z*1) in Fiber 1 at a propagation distance *z*1 can be calculated by

1 $$E1(r,z1)=\sum _{m=1}^{M1}b{1}_{m}\Psi {1}_{m}({\rm{r}})\exp (j\beta {1}_{m}z1)$$

where $\Psi {1}_{m}(r)$ is the field distribution of the Fiber 1, *b*1_*m*_ is the excitation coefficient between fundamental mode of SMF and each mode of Fiber 1, *β*1_*m*_ is the propagation constant of each eigenmode of the Fiber 1.

As light travels from Fiber 1 to Fiber 2, the field $E2(r,z2)$ in Fiber 2 at a propagation distance *z*2 can be calculated by^18^

2 $$E2(r,z2)=\sum _{m=1}^{M2}b{2}_{m}{\rm{\Psi }}{2}_{m}({\rm{r}})\exp (j\beta {2}_{m}z2)$$

where $\Psi {2}_{m}(r)$ is the field distribution of the Fiber 2, *b*2_*m*_ is the excitation coefficient between overall field of Fiber 1 and each mode of Fiber 2, *β*2_*m*_ is the propagation constant of each mode of the Fiber 2.

At the end of both Fiber 1 and Fiber 2, the lights reflected back to the input SMF. The field distribution at the input SMF can be expressed as:

3 $$E(r,z)=\{1+\sum _{m=1}^{M2}b{2}_{m}{\rm{\Psi }}{2}_{m}({\rm{r}})\exp (j2\beta {2}_{m}z2)\}\sum _{m=1}^{M1}b{1}_{m}{\rm{\Psi }}{1}_{m}({\rm{r}})\exp (j2\beta {1}_{m}z1)$$

Eq. (3) shows that the length of Fiber 1 (*z*1) determines the envelope of the filter and the length of Fiber 2 (*z*2) determines the channel spacing of the filter.

The transmission power at the output SMF can be determined by using overlap integral method between *E*(*r*, z) and the fundamental mode of the output SMF $\phi (r)$ as

4 $$L=10\cdot lo{g}_{10}[\frac{{|{\int }_{0}^{\infty }E(r,z)\phi (r)rdr|}^{2}}{{\int }_{0}^{\infty }{|E(r,z)|}^{2}rdr{\int }_{0}^{\infty }{|\phi (r)|}^{2}rdr}]$$

By solving Eq. (4), the transmission of the structure can be determined.

Due to the introduction of misalignment between Fiber 1 and 2, the combined fiber structure section will be birefringent and hence the fiber comb filter is expected to be polarization dependent. To analyze the polarization properties of two-beam fiber comb filter we will consider it as a Mach-Zehnder interference. Consider the Poincare sphere representation^24^ of the state of polarization (SOP) of the light at the input and in Fiber 1 (*C*_1_) and Fiber 2 (*C*_2_) of the interferometer at the point of recombination. The output fringe visibility is simply given by^25^

5 $$V=\,\cos \,\eta $$

where 2*η* is the angle subtended by the great circle are *C*_1_*- C*_2_ at the center of the sphere. The coordinate of *C*_1_ and *C*_2_ depend on the polarization evolution along the two arms of the interferometer and *C*_*i*_. The polarization evolution of the input state, *C*_*i*_, along Fiber 1 and Fiber 2 is described by Poincare sphere operators ${ {\mathcal R} }_{1}$(${\Omega }_{1}$) and ${ {\mathcal R} }_{2}$(${\Omega }_{2}$), respectively. The input state *C*_*i*_ is transformed into different states *C*_1_ and *C*_2_, and an input state coincident with the eigenmodes of ${ {\mathcal R} }_{1}$ or ${ {\mathcal R} }_{2}$ remains invariant in the polarization evolution of *C*_*i*_ to *C*_1_ or *C*_*i*_ to *C*_2_, respectively. The interferometer output at the point of recombination of the two beams is viewed in a frame of reference rotated by ${ {\mathcal R} }_{1}^{-1}$. In the frame Fiber 1 appears isotropic (net operation $\,{ {\mathcal R} }_{1}^{-1}{ {\mathcal R} }_{1}=1$), whereas Fiber 2 operator is ${ {\mathcal R} }_{2-1}{{\rm{\Omega }}}_{2-1}=\{{ {\mathcal R} }_{1}^{-1}{ {\mathcal R} }_{2}\}$. The operator can be used to express analytically the visibility of the structure in term of the input SOP.

For an arbitrary input SOP, *C*_*i*_, the angular shift imparted in *C*_*i*_ by ${ {\mathcal R} }_{2-1}$ is given by spherical geometry according to^26^

6 $$\eta =si{n}^{-1}[sin\theta \,\sin ({{\rm{\Omega }}}_{2-1}/2)]$$

where *θ* and ${\Omega }_{2-1}\,$are the angle subtended by the great circle arc ${C}_{i}-{ {\mathcal R} }_{2-1}$ on the input Poincare sphere and the differential birefringence between two fibers, respectively. Based on Eq. (5), the visibility can thus be expressed as

7 $$V={[1-si{n}^{2}\theta si{n}^{2}({{\rm{\Omega }}}_{2-1}/2)]}^{1/2}$$

Here, ${\Omega }_{2-1}\,$is a unique parameter of the structure. Therefore, the output spectrum visibility depends on input SOP, *C*_*i*_.

**Figure 4:**
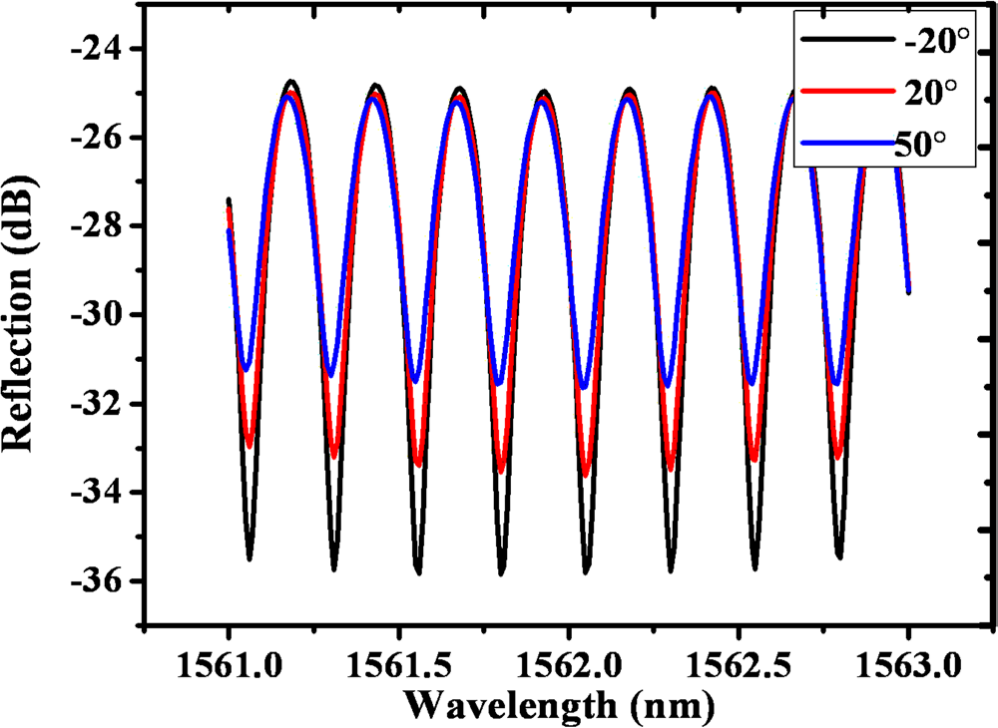
The measured spectral responses for the new structure (Fiber 1: NC125, 7.0 mm; Fiber 2: ANDREW55, 3.3 mm) at different input linear polarizations.

In order to investigate the polarization dependence of the proposed comb filter, an adjustable PC was employed before connecting the input light to Port 1 of the circulator. Figure 4 shows the measured polarization dependent spectral responses of a sample fiber filter made by fusion splicing an SMF with a short section of Fiber 1 (7.0 mm long NC 125) followed by Fiber 2 (3.3 mm long ANDREW55, which has fiber diameter of 55 µm made by ANDREW). The sample was prepared by the following steps: firstly, a short section of Fiber 1 was fusion spliced with a conventional SMF, and then Fiber 2 was fusion spliced with Fiber 1 with a suitable misalignment to achieve equal reflectivity from both the end faces of Fiber 1 and 2; secondly, the side surface of the new structure was coated with a polymer film; finally, a gold film was coated on the end surfaces of both, Fibers 1 and 2, with a sputter machine, followed by removal of the polymer from the side surface.

**Figure 5:**
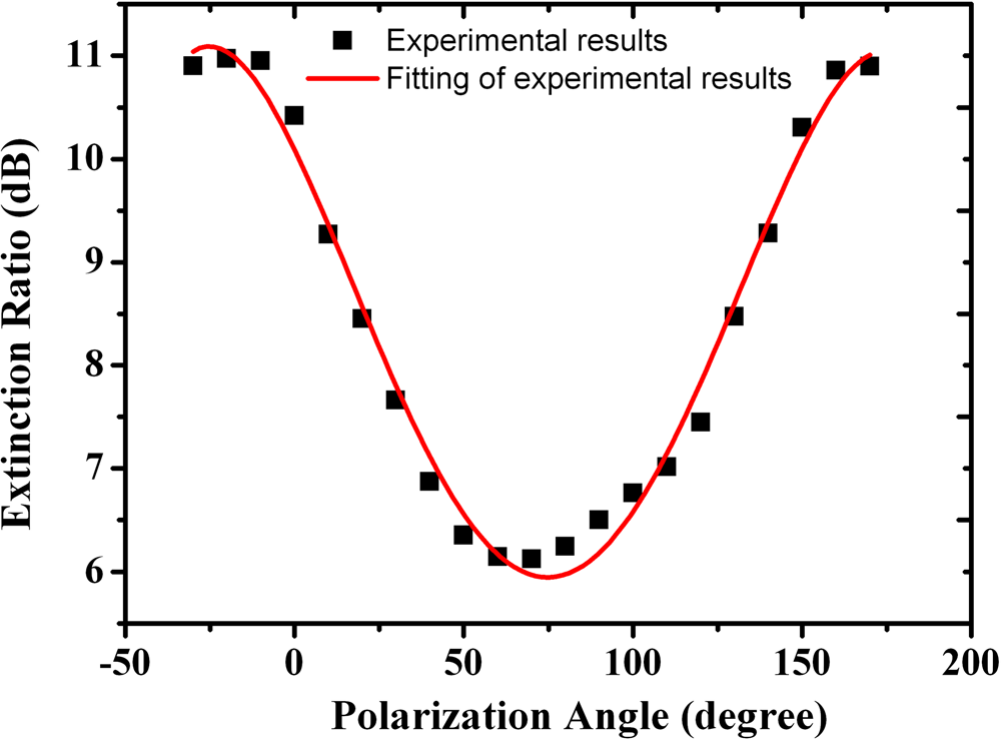
The measured extinction ratios at different polarization angles. The extinction ratio varies from 6 to 11.1 dB.

As shown in Fig. 4, firstly the FSR remains unchanged at different SOPs of the input light; secondly, the extinction ratio of the spectral response is dependent on SOP of the input light, which verified our prediction above in Eq. (6). Figure 5 illustrates the extinction ratios measured at different linear SOPs varied from −30° to 180° with a step of 10°. The minimum and maximum extinction ratios of 6 dB and 11 dB were observed, at SOPs of 75°, and −26°, 170° respectively.

**Figure 6:**
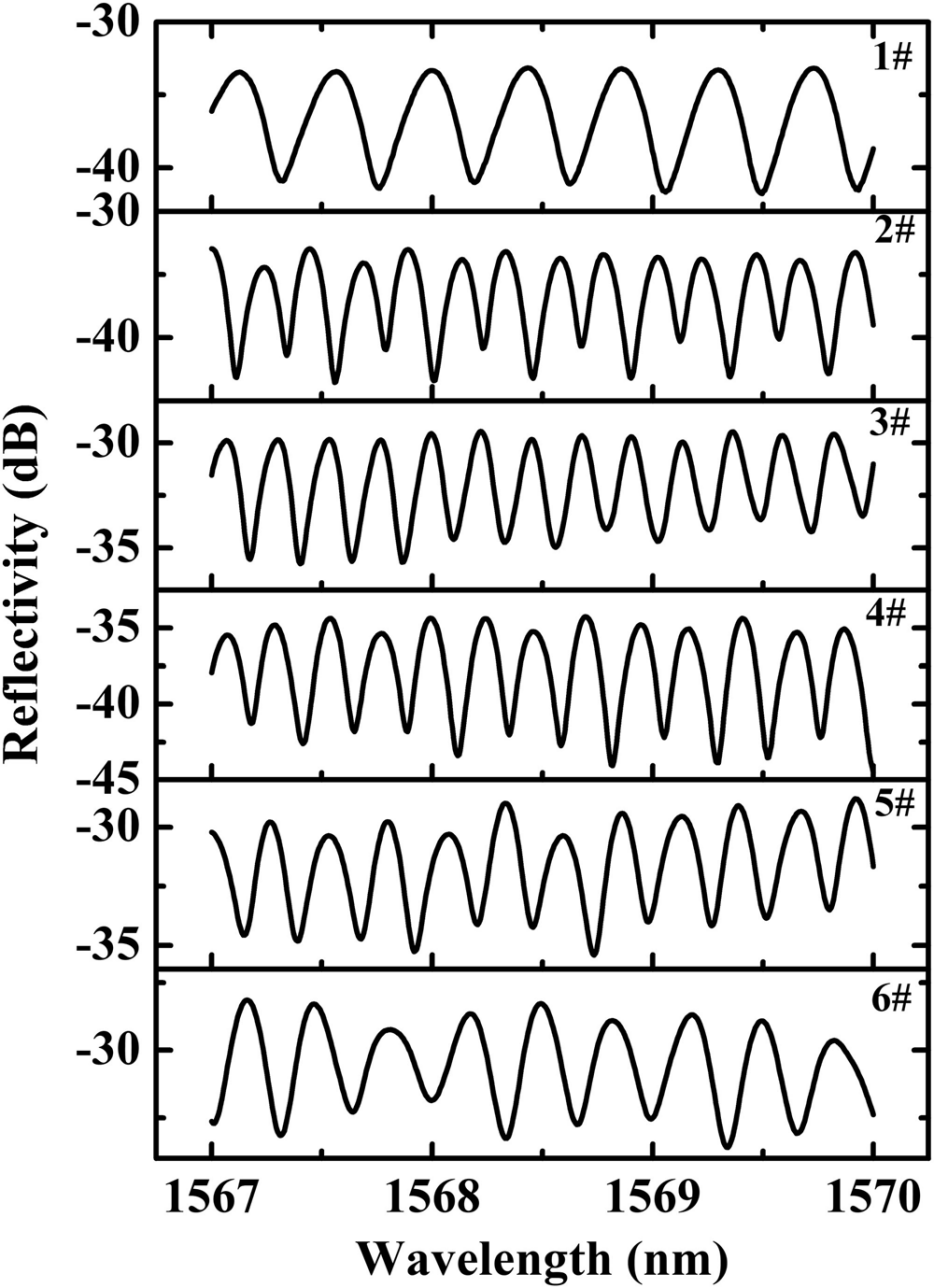
The spectral response of different lengths of fiber 1 and 2 for the proposed comb filter structure.

In order to investigate the influence of fiber length of fiber 1 and 2 on the free spectral range (FSR) of the structure, experiments were firstly carried out using different lengths of fiber 1 and 2. The spectral response of those samples is shown in Fig. 6 and the summary result is shown in Table 1. The fiber 1 and 2 used in this test are NC125 and NC61.5 respectively. In the preparation of the sample, the cutting of the end face of the fiber 2 was implemented by a custom-made fiber cleaver. At the same time, the micro-manipulation for the fiber 2 was implemented under stereomicroscope (Nikon SMZ1500) in order to control the length of fiber 2.

**Table 1:** Different lengths of fiber 1 and 2 for the proposed comb filter structure.

Sample Number	Fiber 1 (NC125) Length (mm)	Fiber 2 (NC61.5) Length (mm)	FSR (nm)
1	1.9	2.0	0.455
2	1.9	3.8	0.227
3	3.9	3.7	0.230
4	2.2	3.6	0.235
5	1.6	3.2	0.243
6	3.2	2.5	0.303

Table 1 shows that with same length of Fiber 1 (Sample 1 and 2), the length of Fiber 2 has significant influence on the channel spacing. Sample 2, 3 and 4 have similar length of Fiber 2 but different length of Fiber 1, and they have similar FSR, indicating that the length of Fiber 1 has limited influence on the channel spacing of the filter. Since optical path differences between reflected light at Fiber 1 and Fiber 2 could change the spectral spacing of the output light, the length of Fiber 2 dominates the FSR, the longer the fiber 2, the smaller the FSR.

**Figure 7:**
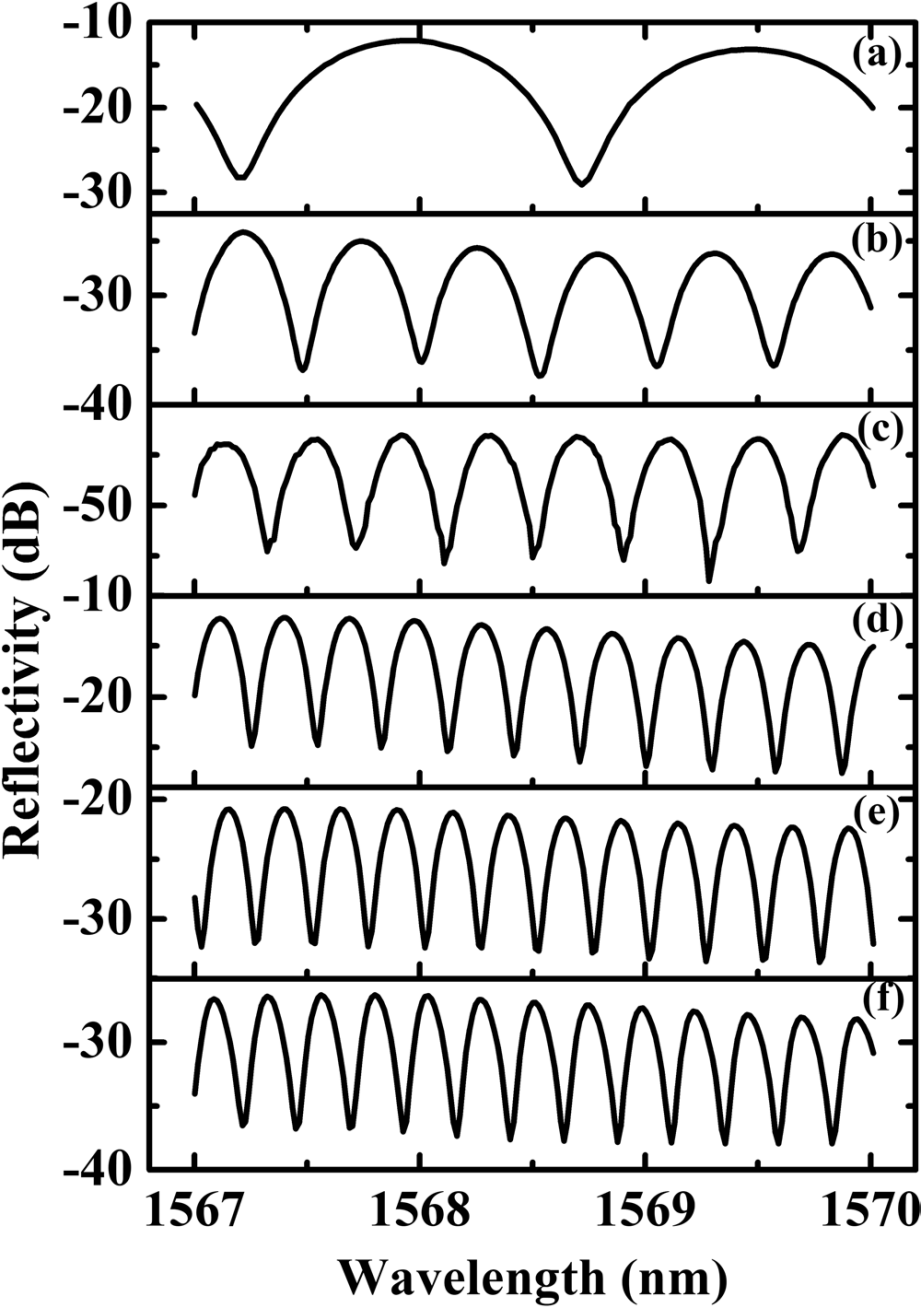
Experimental measured reflectivity curves of the comb filter, the spectral evolved from 0.236 nm to 1.524 nm. (**a**) 1.524 nm. (**b**) 0.521 nm. (**c**) 0.397 nm. (**d**) 0.283 nm. (**e**) 0.254 nm. (**f**) 0.236 nm. The different insertion losses of each sample may lead to different y-axis scale.

In order to investigate the influence of different fiber types and lengths on the spectral response of the comb filter, six samples with different fiber types and lengths were prepared and experimentally investigated. During the fabrication process, in order to make the predesign misalignment between Fiber 1 and 2 easily, large-core multimode fiber or no-core fiber was selected as Fiber1. On the other hand, In order to reduce the coupling loss between Fiber 1 and 2, small cladding diameter fiber was chosen as Fiber 2. According to Eq. (3), the length of Fiber 1 and 2 determines the general loss and FSR of the structure respectively. In the experiments, we selected same length of Fiber 1 but different length of Fiber 2, and same length of Fiber 2 but different length of Fiber 1 to verify our predictions. The reflective spectral responses of the six samples are shown in Fig. 7. The detailed fiber types and lengths used in the experiments are listed in Table 2. The spectral responses indicate that there is difference of the extinction ratio at different SOPs. In the experiment, the extinction ratio is defined as *EX* = *I*_*max*_ − *I*_*min*_^27^, where *I*_*max*_ and *I*_*min*_ represent the adjacent maximum value and minimum value of the spectral responses respectively. The extinction ratio as one of important performance parameters of the fiber comb filter was calculated through the definition mentioned-above in Table 2. As can be seen from Fig. 7, the FSR varies from 0.236 nm to 1.524 nm with different lengths and types of Fibers 1 and 2, which confirmed the conclusions above that the length of Fiber 2 dominates the FSR. From Fig. 7, the minimum insertion loss of the proposed comb filter structure is about 12 dB. This is mainly due to the contribution of Fiber 1 to the envelope of the filter as shown in Eq. (3). According to Eqs (3) and (4), if both the length of Fiber 1 and 2 can be optimized, the insertion loss can be reduced significantly.

**Table 2:** Types and lengths of fibers for the proposed comb filter structure.

Sample Number	Fiber 1		Fiber 2		Extinction Ratio(dB)
	Material	Length (mm)	Material	Length (mm)	
a	AFS105/125	4.0	ANDREW55	0.6	17.01
b	AFS105/125	5.3	ANDREW55	1.6	12.06
c	NC61.5	20.1	ANDREW55	2.1	11.15
d	AFS50/125	8.3	ANDREW55	3.0	12.75
e	NC125	7.0	ANDREW55	3.3	11.51
f	SM460	7.2	SM460	3.6	10.17

**Figure 8:**
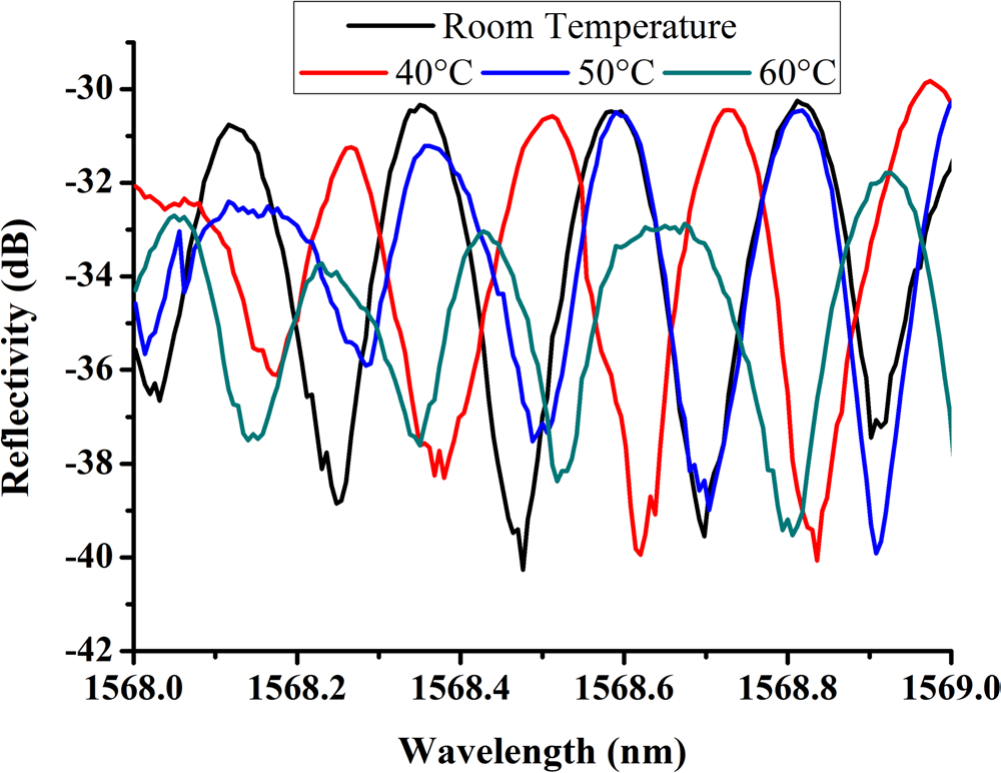
The measured spectral responses for Sample 3 (Fiber 1: NC125, 3.9 mm; Fiber 2: NC61.5, 3.7 mm) at different surrounding temperature.

For the development of the proposed fiber comb filter structure in communication system application, it is significant to study its thermal stability. In the experiment, Sample 3 was fixed on a manual control lifting platform to get close slowly to a magnetic stirrer with the function of precise control of temperature (IKA RET/T). In order to keep the temperature consistency between the stirrer and Sample 3, the sample should be close to the metal heating plane of the stirrer as possible (about 0.5 mm). The spectral responses of Sample 3 at different surrounding temperature are illustrated as Fig. 8.

From Fig. 8, it is noted that the FSR shift caused by surrounding temperature of Sample 3 decreases gradually with the temperature increasing, but not obvious. In Fig. 8, it can be seen that the insertion loss shift resulted from temperature is far less than the insertion loss of the device, which agrees well with the results in reported ref.^28^. Although FSR shift caused by surrounding temperature is not obvious, it is critical to avoid the large variation of surrounding temperature of the device employing the combined multimode interference and Mach-Zehnder interferometer method due to distortion of spectrum.

## Conclusion

In conclusion, we have experimentally demonstrated an all-fiber comb filter based on the combined effect of multimode interference and Mach-Zehnder interferometer. This comb filter was made by fusion splicing of a short section of MMF to a conventional SMF, followed by a short section of other type of fiber with a designed misalignment. Experimentally we have achieved different FSRs by using different types and lengths of the fiber sections. This comb filter is polarization dependent and experimentally its extinction ratio varies from 6 to 11.1 dB. The proposed all-fiber comb filter has the advantages of simple fabrication and compact design. It has potential applications in optical fiber communication systems, such as light signals filtering in DWDM system.
